# The Evaluation of Construction Dust Diffusion and Sedimentation Using Wind Tunnel Experiment

**DOI:** 10.3390/toxics10080412

**Published:** 2022-07-22

**Authors:** Yisheng Zhang, Wei Tang, Hao Li, Jinjun Guo, Jingjiang Wu, Yongfu Guo

**Affiliations:** 1 School of Water Conservancy and Civil Engineering, Zhengzhou University, Zhengzhou 450001, China; yishengzhang@zzu.edu.cn (Y.Z.); tangwei251231@163.com (W.T.); haol950@163.com (H.L.); 2 Yellow River Laboratory, Zhengzhou University, Zhengzhou 450001, China; 3 China Construction Seventh Engineering Bureau Co., Ltd., Zhengzhou 450012, China; wujingjiang@cscec.com (J.W.); guoyongfu1@cscec.com (Y.G.)

**Keywords:** particulate matter, wind tunnel experiment, moisture content, peak concentrations, particle inhibition rate, spread distance

## Abstract

A large quantity of particulate matter is generated during construction of civil engineering projects, which has a negative effect on the atmosphere and environment. In order to explore the concentration, distribution and diffusion of particulate matters generated from construction dust with different moisture contents, a wind tunnel experiment was conducted, and the effects of wind speed and moisture content on the inhibition rate, drifting distance and suppression percentage of particulate matters were investigated. The results show that the peak concentration decreases with the increase in moisture content, compared with dry dust; the peak concentrations for 1%, 2% and 3% moisture content are reduced by 37.07%, 39.53% and 65.38%, respectively. The average concentrations in the cross-section decrease with the increase in the moisture content, resulting in an increasing tendency of the particle inhibition rate. The forecast drifting distance decreases with the increase in the moisture content; when the suspension percentage is 1%, the forecast drifting distances of dry dust, 1%, 2% and 3% moisture content are 641.58, 116.08, 19.33 and 3.82 km, respectively, for a 5 m/s wind speed. Considering that an increase in wind velocity will not only decrease the inhibition rate but also increase the drifting distance, the dust suppression method by increasing the moisture content in low and medium wind velocities is applicable. When the limit value of the particle suppression rate within a distance of 50 m is larger than 70%, construction activities are prohibited at any wind velocity for dry and 1% moisture content, and at wind velocities larger than 2 m/s and 4 m/s for 2% and 3% moisture content, respectively.

## 1. Introduction

In China, the number of construction projects increased rapidly with the urbanization process [1]. During the construction of civil engineering projects, a large quantity of particulate matter is generated, which has a negative effect on the atmosphere and environment [2,3]. Many researches have shown that particle pollution caused by civil engineering construction has become the primary air pollution in China [4,5]. As a result, air pollution of particulate matter caused by civil engineering is the fourth most common factor to threaten people’s health next to nutrition, hypertension and smoking [4]. Therefore, with the aim to improve people’s quality of life, investigating the drift–diffusion characteristics of dust, and reducing the emissions of particulate matter during civil engineering construction projects is of great significance.

In the early 1990s, Tharr et al. [6] investigated construction dust exposure for dust-producing construction tasks. Since then, many scholars have explored the effective treatment to deal with construction dust and obtained some desirable achievements. For example, Tomb et al. [7] used experimental methods to study and determine the effect of spray water droplets on dust suppression; the result showed good dust suppression efficiency under the best spray conditions. Similar results were obtained by Zhou et al. [8] and Guo et al. [9]. However, these researches mainly focused on spray atomization, which increases the air humidity to increase the probability of coalescence among particles, so as to analyze the dust suppression effect as it is difficult to mitigate construction dust particles when they are fully spread. Zhu et al. [10] conducted a series of experiments of dust diffusion using numerical simulation and field tests. By monitoring the concentration of particles near the expressway, Imhof et al. [11] found that the concentration of particles increased first and then decreased with the increase in height. Different to Imhof et al. [11], Tian et al. [12] presented a different conclusion by collecting dust particles from different heights near the construction site: the dust’s concentration was inversely proportional to the square of the height. In order to study the effects of environmental factors on the distribution of dust, Neuman et al. [13] explored the vertical distribution of dust with different wind speeds using a wind tunnel experiment; the results showed that wind speed has an obvious effect on the diffusion of particulate matter, and the concentration of fugitive dust decreases with the increase in drift distance. After this, Amato et al. [14] analyzed the emissions of particulate matter on paved roads using an experimental method; the result showed that the diffusion of dust is affected by the quantity of soil on the road, air temperature, atmospheric moisture, rainfall and so on. More specifically, He et al. [15] measured the concentration of dust particles; the result verified that dust concentration decreases with an increase in the drift distance; in addition, wind speed has a significant effect on the diffusion of dust particles in the vertical direction. Sanderson et al. [16] measured the emission rate of PM10 (particles with diameters <10 μm) from smelter slag with varied wind speeds, combining a field test and a wind tunnel experiment, and demonstrated that the emission rate depends on the particle supply, which declines rapidly with time after exposure to airflow. Furthermore, a series of experiments were carried out by Joanna et al. [17] in the wind tunnel to analyze the effect of temperature and relative humidity on dust emission. For the purpose of providing insights for mitigating dust pollution caused by the construction industry, Wu et al. [18] investigated the prevention of construction dust in China and proposed some policy recommendations. Previous studies have investigated dust diffusion in space using numerical simulation, wind tunnel experiments and/or field tests, and revealed the environmental factors that affect the drift and diffusion of dust. However, it remains unclear how the dust moisture characteristic affects the dust’s emission under different wind conditions.

Hence, this study aims to investigate the concentration distribution and diffusion of particulate matters generated from dust with different moistures by a laboratory experiment. On this basis, the inhibition rate and spread distance of PM_2.5_ (particles with diameters <2.5 μm) were calculated under different wind speeds, and the effects of wind speed and dust moisture on mitigating dust pollution were analyzed.

## 2. Materials and Methods

### 2.1. Indoor Experiment Setup

**Figure 1:**
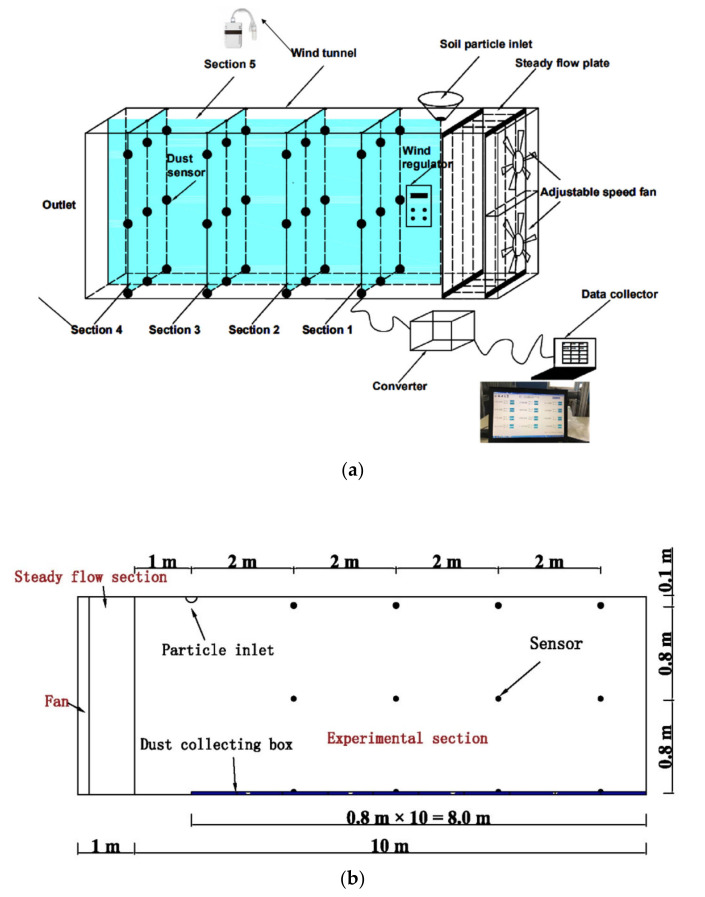
Experimental setup for particulate matter diffusion. (**a**) Model of wind tunnel equipment, (**b**) front view of wind tunnel equipment.

The experiment was conducted at the water Hydraulics Laboratory of Zhengzhou University in China. The indoor experiment platform consists of the adjustable speed fan, the steady flow plate, the wind tunnel, the dust sensors, the data converter and the data collector. The wind tunnel length, width and height are 11 m, 1 m and 2 m, respectively. The airflow was generated by a fan system precisely controlled by regulator. The wind speed ranged from 1 to 5 m/s. In order to stabilize the airflow, the equipment consisting of steady flow plate was settled in the front of wind tunnel. The model of wind tunnel equipment is illustrated in Figure 1a,b, showing the size of the equipment. As shown in Figure 1, particle inlet was set directly above the steady flow section. The steady flow section consisted of several fixed plates, which made the wind as steady as possible. Laser dust sensors (range from 0 to 1000 μg/m^3^) were adopted to measure the concentration of particulate matter in experimental section. In order to analyze the dispersion of particulate matter along the flow direction, ten dust collection boxes were installed at the bottom of the experimental section. A high precision analytical balance was utilized to quantify the mass of fallen dust.

### 2.2. Experimental Method

**Figure 2:**
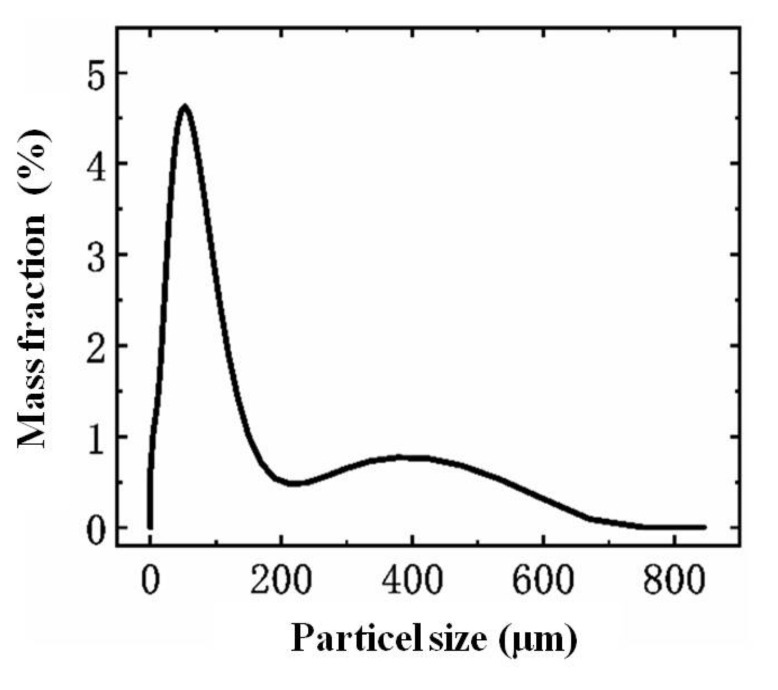
Particle size distribution.

**Figure 3:**
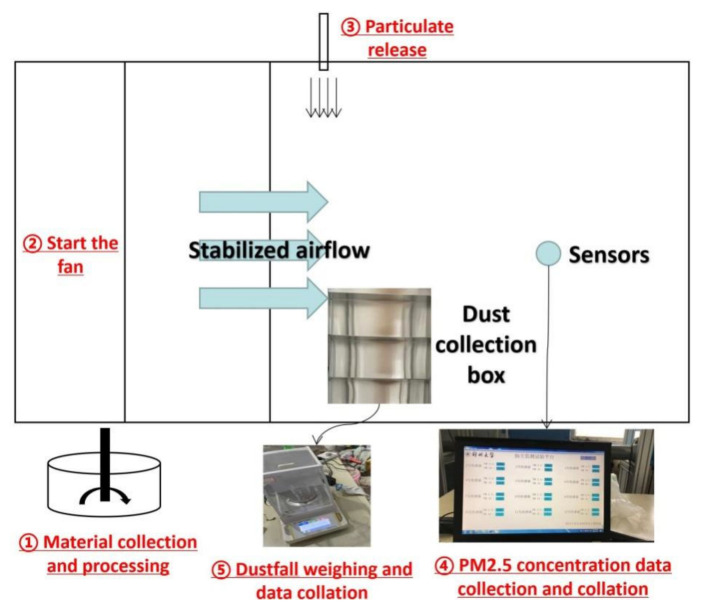
Schematic diagram of experimental procedure.

Experimental procedure was divided into four parts: dust preparation, fan system initializing, particle released and data collation. Experimental dust was taken from a construction site in Zhengzhou city; the particle size distribution of the test particles is shown in Figure 2. The dust sample was dried in an oven at 150 °C for 5 h and then put into a sealed box to cool down. In order to investigate the influence of moisture content on dust concentration distribution and diffusion, the volume moisture content of the test dust was dry, 1%, 2% and 3%. After the dust preparation procedure, the fan system was activated to adjust the wind velocity. Wind speed was measured by an anemometer, in order to investigate the influence of wind speed on dust drift and diffusion; the test turbulent flow was set as 1, 2, 3, 4 and 5 m/s. Specific experimental condition design is shown in Table 1. Then, 100 g dust was released at a constant speed with settled moisture content within 1 min, and the dust concentration collection software was applied to measure the concentration. After the test, the dust in collection boxes was weighed. All the experiments were repeated for three times, and the average value was analyzed in each working condition. The experimental step is shown in Figure 3.

### 2.3. Assessment Method

(1) Particle inhibition rate

The particle inhibition rate was introduced to quantify the suppressed relationship between moisture content and mean PM_2.5_ concentration in the observed section, and the particle inhibition rate can be listed as

(1) $$P_{i}=\frac{\bar{C_{0}}-\bar{C_{i}}}{\bar{C_{0}}}$$

where: $P_{i}$ is the average particle inhibition rate when the water content of particles is i, %; $\bar{C_{0}}\;$ is the average concentration for dry dust, g/m^2^; $\bar{C_{i}}$ is the average concentration when the moisture content of particles is i %, g/m^2^.

(2) Mass of drift losses

In order to analyze the diffusion of particulate matter along the flow direction, this paper introduces the concept of drift losses, which is calculated as:

(2) $$M_{x}=M_{0}-M_{x}^{\prime }$$

where: $M_{x}$ is the total mass of drift losses beyond the x m, g; $M_{0}$ is the initial mass of particles, 100 g; $M_{x}^{\prime }$ is the total dustfall mass within the x m, g.

(3) Suspension percentage

Some particulate matter stays partially in the air during the diffusion process; in order to evaluate the dust suspension percentage, suspension percentage was introduced as

(3) $$P_{x}=\frac{M_{x}}{M_{0}}$$

where $P_{x}$ is the dust suspension percentage beyond x m, %.

(4) Diffusion distance

The drifting distance is inversely proportional to drifting mass under different wind velocities; hence, an inverse proportional function model was established as follows

(4) $$M_{x}=\frac{a}{x^{b}}$$

where *x* is the drifting distance, m; *a*, *b* are the diffusion coefficients.

As defined in Equations (2) and (3), the drift mass of particles can also be expressed by

(5) $$M_{x}=M_{0}\times P_{x}$$

The diffusion distance can be derived by Equations (4) and (5) as below

(6) $$x=\sqrt[{b}]{\frac{a}{M_{0}\times P_{i}}}$$

(5) Particle suppression efficiency

Particle suppression efficiency within *x* m is expressed as follows

(7) $$Se_{x}=\frac{M_{x}^{dry}-M_{x}^{n}}{M_{x}^{dry}}\times 100\%$$

where $Se_{x}$ is suppression efficiency within *x* m distance; $M_{x}^{dry}$ is mass of drift losses beyond *x* m distance for dry dust, g; $M_{x}^{n}$ is the mass of drift losses beyond *x* m distance for *n* moisture content, g.

## 3. Results

### 3.1. Impact of Moisture on Concentration of PM2.5

**Figure 4:**
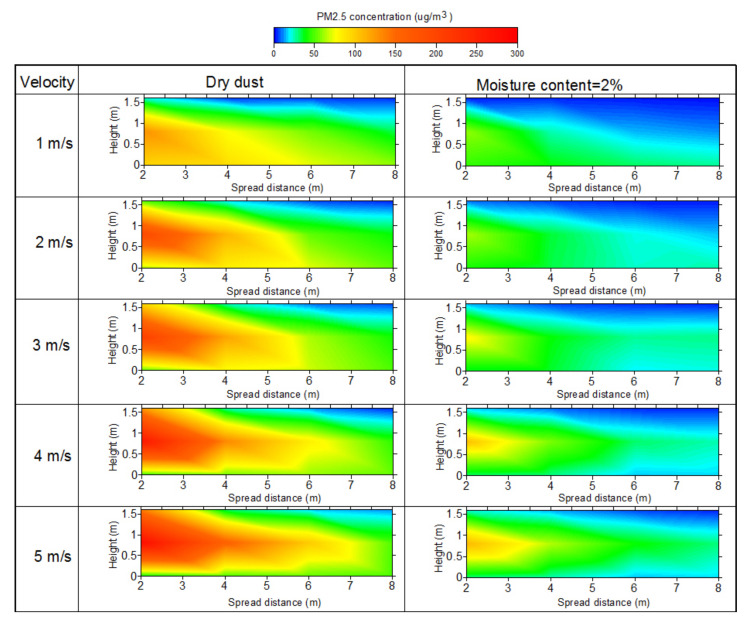
Contour of PM_2.5_ concentration distribution (μg/m³).

The monitoring surface of the center section in the flow direction was selected to analyze PM_2.5_ concentration distribution in this paper under different wind velocities. The data collected from each point were integrated and the mass concentration contours were plotted based on Kriging interpolation using Surfer software (version: Surfer 8.0). Figure 4 presents the contours of the concentration distribution of PM_2.5_ for dry and 2% moisture content dust. For constant moisture content, the concentration of PM_2.5_ increased with the increase in wind velocity, and the peak concentration appeared in the initial position at the height of 0.8 m.

**Figure 5:**
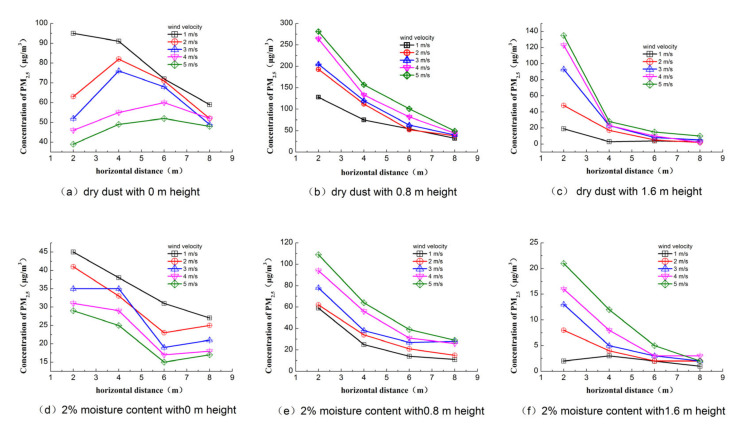
Concentration of PM2.5 along the flow direction.

The variations in PM_2.5_ concentration with dry and 2% moisture content dust for different heights are presented in Figure 5. For dry dust, the peak concentration of PM_2.5_ increased with the decrease in wind velocity at a height of 0 m, and the value showed an increasing tendency and then decreased as the flow distance increased. For the heights of 0.8 and 1.6 m, the peak concentration of PM_2.5_ presented an opposite trend with the increase in wind velocity. The reason for this change is that most particles fell on the ground nearby the source of the dust before they fully spread under low wind velocity, resulting in a high concentration. The PM_2.5_ concentration decreased as the flow distance increased. In addition, the peak concentration increased from 128 μg/m^3^ to 281 μg/m^3^ when the wind velocity increased from 1 m/s to 5 m/s, with an increasing ratio of 119.53%. As the moisture content increased to 2%, the variation in PM_2.5_ concentration was similar to dry dust, but the value was lower than dry dust.

**Figure 6:**
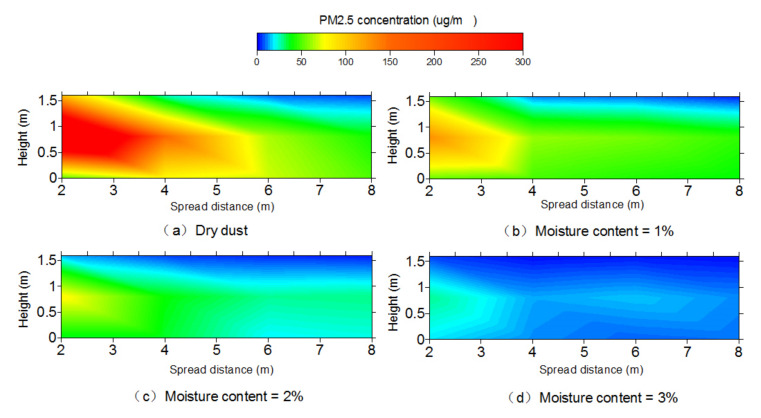
Contours of PM_2.5_ concentration with different moisture contents with 3 m/s wind velocity.

**Figure 7:**
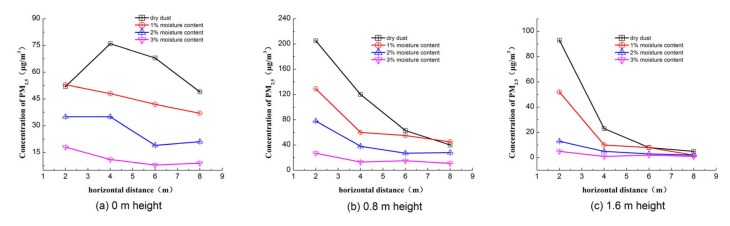
Concentration of PM_2.5_ along the flow direction with 3 m/s wind velocity.

In order to investigate the impact of moisture content on particle diffusion, the concentration distribution of PM_2.5_ with different moisture contents under five wind velocities were analyzed. Figure 6 shows the concentration of PM_2.5_ with different moisture contents under a 3 m/s wind velocity. As shown, the peak concentration decreased with the increase in moisture content. For dry dust, 1%, 2% and 3% moisture content dusts, the peak concentrations were 205, 129, 78 and 27 μg/m^3^, respectively, and, compared with dry dust, the peak concentrations for 1%, 2% and 3% moisture contents were reduced by 37.07%, 39.53% and 65.38%, respectively. Figure 7 shows the concentration value along the flow direction with different heights. The concentration of PM_2.5_ decreases with the increase in moisture content at the same position. It indicates that increasing the moisture content through the spraying of water can inhibit the peak concentration effectively, which is similar to the conclusion obtained by other scholars [19].

### 3.2. Particle Inhibition Rate

**Figure 8:**
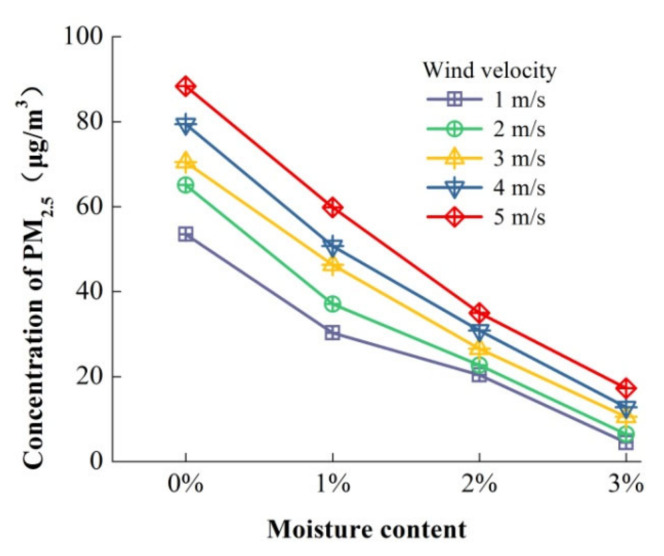
Average concentration of PM_2.5_ in cross-section.

As shown by the analysis above, increasing the moisture content of particulate matter contributes to dust suppression. To further study the effect of the moisture content of particles on dust diffusion, the average concentration and the particle inhibition rate of PM_2.5_ on the cross-section under different wind velocities were analyzed. As shown in Figure 8, the average concentration in the cross-section decreased with the increase in the moisture content with any wind velocity.

**Figure 9:**
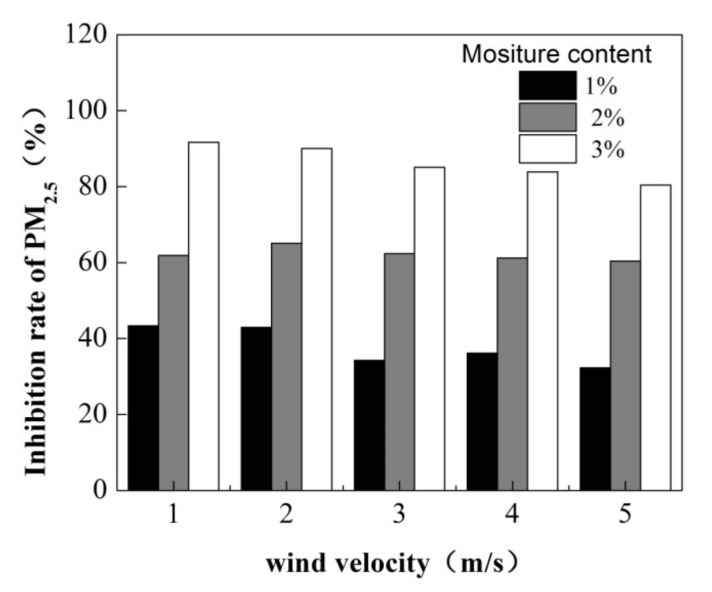
Particle inhibition rate of PM_2.5_.

Figure 9 shows the particle inhibition rate with different moisture contents. As shown in Figure 9, the particle inhibition rate was larger than 30%, 60% and 80% under different wind velocities as the moisture content increased to 1%, 2% and 3%, respectively. It demonstrates that when the moisture content of a particle increases, the particle inhibition rate is higher, i.e., the increase in the moisture content of a particle has a significant influence on the dust drifting. In particular, when the moisture content was 3%, the inhibition rates were 91.67%, 90.08%, 85.07%, 83.90% and 80.44%, respectively, as the wind velocity ranged from 1 to 5 m/s. Figure 9 also shows that the inhibition rate under the same moisture content tended to decrease with the increase in wind velocity. In particular, when the moisture content was 3%, the inhibition rate decreased from 92% to 80% as the wind velocity increased from 1 m/s to 5 m/s. It indicates that moisture content should be increased under high wind velocity during the construction process.

### 3.3. Prediction of Spread Distance

**Figure 10:**
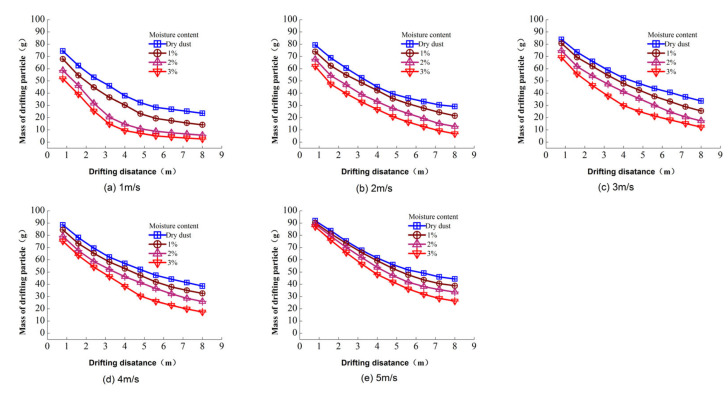
Variations in the drift losses mass of particulate matter.

In order to analyze the particle diffusion distance along the flow direction, the mass of drifting particles with different moisture contents under a 5 m/s wind velocity were calculated. As shown in Figure 10, when the moisture content increased from 0% to 3%, particle drift losses beyond 8 m reduced by 89.43%, 76.78%, 63.5%, 54.83% and 41.69% for the wind velocities 1, 2, 3, 4 and 5 m/s, respectively; it indicates that increasing the moisture content could have a significant inhibition effect on the horizontal drifting distance.

The experimental data with the different moisture contents and wind velocities were applied for nonlinear fitting, and the values of *a* and *b* in each inverse proportional function were obtained; the results are shown in Table 2, and *R^2^* is the coefficient of determination.

The coefficients of determination in Table 2 are higher than 0.9 under different working conditions; it demonstrates that the drifting distance can be predicted by Equation (6).

**Figure 11:**
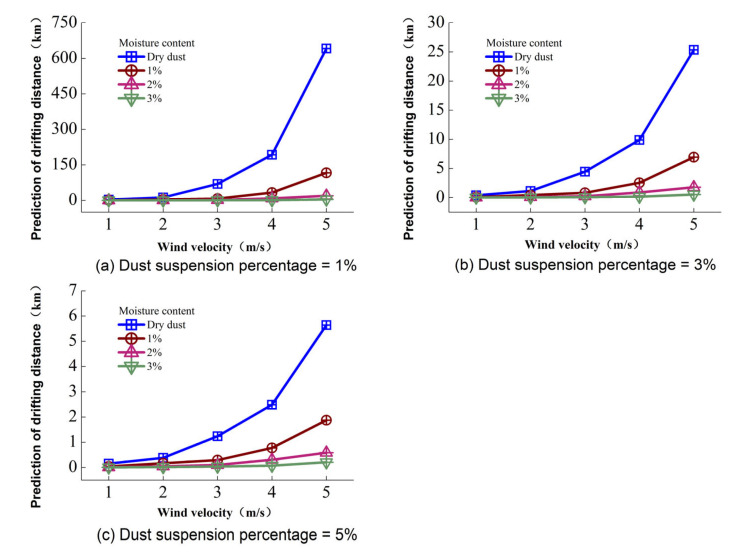
Particle diffusion distance with different suspension percentages.

Considering some particles were unable to land within a specific distance, 1%, 3% and 5% dust suspension percentages were applied to analyze the influence of moisture content on drifting distance. Figure 11 is the prediction of the particle dispersion distance with different suspension percentages under different wind velocities. Figure 11 shows that the particle drifting distance decreased with the increase in the moisture content for a permanent wind speed at any dust suspension percentage; for example, when the suspension percentage was 1%, the drifting distances of the dry dust, 1%, 2% and 3% moisture content dusts were 641.58, 116.08, 19.33 and 3.82 km, respectively, with a 5 m/s wind speed. Figure 11 also shows that wind speed has a direct effect on the drifting distance; a long drifting distance can be obtained with a high wind speed. When the suspension percentage was 5%, the drifting distances for dry dust were 0.15, 0.38, 1.24, 2.48 and 5.91 km as the wind velocity increased from 1 to 5 m/s, respectively.

## 4. Discussion

Particle suppression efficiency is used to analyze the effect of moisture content on the particle diffusion. The particle suppression efficiency within a distance of 50 m was calculated by Equation (7) combined with Equation (4); the results are shown in Table 3. Increasing the moisture content of particles could effectively improve the suppression efficiency. Suppression efficiency increased from 54.87% to 97.26% as the moisture content increased from 1% to 3%, respectively, for a 1 m/s wind velocity. Suppression efficiency decreased when the wind velocity increased; in particularly, when the wind velocity increased from 1 to 5 m/s, a large decrease in the suppression efficiency from 97.26% to 56.47% was obtained for a moisture content of 3%. It indicates that the dust suppression method by increasing the moisture content is effective. For example, when the limit value of the particle suppression rate is larger than 70%, construction activities are prohibited at any wind velocity for dry and 1% moisture content, and at wind velocities larger than 2 m/s and 4 m/s for 2% and 3% moisture content, respectively.

In this research, it is shown that dust can drift for several kilometers to a certain extent, and this value can be calculated by Equation (6). The predicted drift distance has uncertainty because of the diffusion coefficients a and b that are fitted from the curves presented in Figure 10. Despite the coefficient of determination being larger than 0.9, the calculation error still exists; however, the result is similar to that obtained by Zhao [20] and Katra [21]. It means that the calculated drift distance is reasonable. In particular, the flow simulated in a wind tunnel is different from that in the free stream of the atmosphere in reality, and the effects of saltation, atmospheric stability and local convective mixing are not take into account, leading to substantial changes in the near-surface air velocity and direction. Nevertheless, similarly to other research [21], wind tunnel experiments offer valuable insights into the turbulent flow structure under controlled conditions. They help to assess the capacity of quantification of dust processes and support climate models. In addition, vertical velocity is generated during the diffusion in the free stream of the atmosphere due to the effects of atmospheric saltation and the underlying topography, so the particle spread distance may be longer than is predicted in this paper.

## 5. Conclusions

In this paper, a series of experiments on construction dust diffusion were conducted at the Hydraulics Laboratory of Zhengzhou University using a self-designed wind tunnel. The concentration distributions of particulate matter with different moisture contents and wind velocities were investigated. Moreover, the effect of moisture content on the inhibition rate and drifting distance was analyzed.

(1) The moisture content of dust and wind velocity have direct effects on the concentration distribution of PM2.5. The concentration of PM2.5 increased with the increase in wind velocity, and the peak concentration appeared in its initial position at the height of 0.8 m. The peak concentration decreased with the increase in moisture content; compared with dry dust, the peak concentrations for 1%, 2% and 3% moisture content were reduced by 37.07%, 39.53% and 65.38%, respectively.
(2) The average concentrations in the cross-section decreased with the increase in the moisture content, resulting in an increasing tendency in the particle inhibition rate. When the moisture content was 3%, the inhibition rate was 91.67%, 90.08%, 85.07%, 83.90% and 80.44 as the wind velocity increased from 1 to 5 m/s, respectively.
(3) Considering some particles were unable to land within a specific distance, dust suspension percentages of 1%, 3% and 5% were applied to analyze the influence of moisture content on the diffusion distance. The results show that the forecast of the particle dispersion distance decreases with the increase in the moisture content. When the suspension percentage was 1%, the diffusion distances of dry dust, 1%, 2% and 3% moisture content were 641.58, 116.08, 19.33 and 3.82 km, respectively with a 5 m/s wind speed.
(4) Increasing the particulate moisture content can reduce the particulate diffusion; therefore, the dust should be irrigated before construction. Considering the increase in wind velocity not only decreases the inhibition rate but also increases the diffusion distance, the dust suppression method by increasing the moisture content of particles in low and medium wind velocity conditions is applicable, while construction activities under high wind conditions are prohibited.

## Figures and Tables

**Table 1:** Experiment factors and levels.

Factors	Value				
Particle moisture content (%)	0	1	2	3	
Wind velocity (m·s^−1^)	1	2	3	4	5

**Table 2:** Values of *a* and *b*.

Wind Velocity	Moisture Content	*a*	*b*	*R^2^*
1 m/s	dry dust	76.17	0.54	0.96
	1%	72.01	0.73	0.94
	2%	63.91	1.11	0.95
	3%	60.09	1.40	0.94
2 m/s	dry dust	81.90	0.47	0.95
	1%	78.57	0.54	0.92
	2%	74.33	0.71	0.91
	3%	72.19	0.92	0.90
3 m/s	dry dust	86.34	0.40	0.94
	1%	85.76	0.50	0.91
	2%	81.96	0.61	0.90
	3%	75.14	0.75	0.93
4 m/s	dry dust	90.19	0.37	0.95
	1%	87.34	0.43	0.94
	2%	82.59	0.49	0.90
	3%	82.38	066	0.92
5 m/s	dry dust	94.29	0.34	0.95
	1%	94.46	0.39	0.93
	2%	93.68	0.46	0.93
	3%	93.33	0.55	0.92

**Table 3:** Suppression rate of particle within 50 m distance.

Moisture Content	Wind Velocity (m/s)				
	1	2	3	4	5
1%	54.87%	27.05%	32.83%	23.42%	17.62%
2%	90.94%	64.51%	58.25%	42.73%	37.87%
3%	97.26%	84.84%	77.87%	70.63%	56.47%

## Data Availability

Not applicable.
